# Xanthones Isolated from *Cratoxylum cochinchinensis* Reduced Oxidative Stress in Periodontal Ligament Stem Cells

**DOI:** 10.3390/ijms241914675

**Published:** 2023-09-28

**Authors:** Nisarat Ruangsawasdi, Nawong Boonnak, Chareerut Pruksaniyom, Pirasut Rodanant

**Affiliations:** 1 Department of Pharmacology, Faculty of Dentistry, Mahidol University, Bangkok 10400, Thailand; nisarat.rua@mahidol.ac.th (N.R.); chareerut.phr@mahidol.ac.th (C.P.); 2 Department of Basic Science and Mathematics, Faculty of Science and digital innovation, Thaksin University, Songkhla 90000, Thailand; nawongb@yahoo.com; 3 Department of Advanced General Dentistry, Faculty of Dentistry, Mahidol University, Bangkok 10400, Thailand

**Keywords:** xanthone compounds, *Cratoxylum cochinchinensis*, antioxidant, periodontal ligament stem cells

## Abstract

Xanthone compounds from *Cratoxylum cochinchinensis* (*C. cochinchinensis*) have demonstrated antioxidant effects and potency in treating many inflammatory diseases. However, the efficiency of the three xanthone extracts isolated from the young fruit of this plant, i.e., two geranyloxy xanthones (F6, F8) and one 1,3,7-hydroxy xanthone (F137), as antioxidants and therapeutics for periodontal disease has not been evaluated. The aim of this study was to investigate the antioxidant effects of three xanthones isolated from *C. cochinchinensis* on periodontal ligament stem cells (PDLSCs) and their osteogenic differentiation. The antioxidant activity of the aqueous extracts was determined using a DPPH assay, and their cytotoxicity was evaluated using an MTT assay. H_2_O_2_ was used to induce intracellular stress, and the scavenging effect of the isolated compounds against reactive oxygen species (ROS) was analyzed with a fluorescence assay. The expression of nuclear factor-erythroid 2-related factor 2 (Nrf2) and heme oxygenase-1 (HO-1) was evaluated, and the effects of the three compounds on PDLSCs osteogenic differentiation were investigated. The isolated compounds reduced both extracellular and intracellular ROS in a dose-dependent manner and induced the expression of Nrf2 and HO-1 in PDLSCs. Under redox conditions, these compounds potentiated PDLSCs osteogenic differentiation. Our study demonstrated that the hydroxy xanthones from *C. cochinchinensis* had antioxidant effects on the Nrf2/HO-1 pathway and might be effective therapeutic substrates for damage prevention and the regeneration of damaged periodontal tissues in periodontitis patients.

## 1. Introduction

Periodontitis is a chronic inflammatory disease that results in tooth loss. The accumulation of bacterial biofilm on the tooth and in the periodontium results in chronic, severe inflammation leading to tooth loss [1]. Studies have demonstrated that overactivation of polymorphonuclear leukocyte function during the phagocytosis of pathogenic bacteria produces large amounts of reactive oxygen species (ROS) and results in periodontium destruction [2].

Periodontal ligament stem cells (PDLSCs) have been identified in the periodontal ligament (PDL) and contribute to periodontal tissue repair by forming a cementum/PDL-like structure. The inflammatory change in the periodontium causes an increase in PDLSCs number; however, their osteogenic differentiation is impaired [3]. Abundant exogenous ROS produced during periodontal disease degrade soft and hard tissue and decrease the efficiency of PDLSCs in forming new bone due to reducing their multi-lineage differentiation potency [4,5]. Nuclear factor-erythroid 2-related factor 2 (Nrf2) has been identified as a transcription factor that promotes and regulates PDLSCs intracellular antioxidative enzymes, such as heme oxygenase-1 (HO-1), which is upregulated after oxidative stress due to high glucose [6], hydrogen peroxide [7], and bacterial lipopolysaccharides [8]. Although PDL cells stimulate intrinsic antioxidants to limit damage by ROS, an imbalance in elevated ROS and antioxidants can occur and is associated with periodontitis [5]. To overcome the effects of ROS, a treatment that can maximize intrinsic antioxidant activity might be a therapeutic intervention for treating various diseases related to oxidative stress and inflammation.

*Cratoxylum cochinchinensis* (*C. cochinchinensis*), Tiew Kliang, is a tree belonging to the Guttiferae family that is widely distributed in several Southeast Asian countries [9]. Phytochemical studies on plants in the Cratoxylum species revealed various bioactive pure compounds, including xanthones, anthraquinones, and triterpines [10,11]. Several studies have demonstrated that xanthone compounds from plants have important biological effects, including antioxidant, anti-tumor, anti-inflammatory, anti-allergy, and anti-microbial effects [12,13]. However, the effects of xanthones on periodontitis have not been reported, and the mechanism underlying the antioxidant activity of xanthones is not fully understood. Furthermore, xanthones’ chemical structure consists of various components, such as aromatic protons, phenolic hydroxyl groups, methoxyl prenyl groups, hydroxyl protons, and oxygenated methine protons, or a dihydrofuran ring on the xanthone aromatic ring that generates xanthone compounds with different potency degrees [14,15,16].

**Figure 1:**
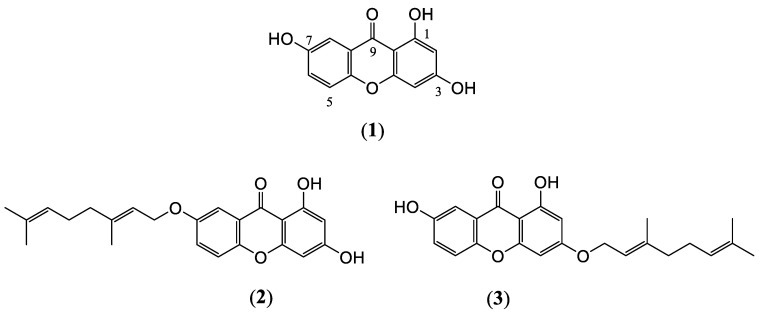
The chemical structure of the xanthones isolated from *C. cochinchinensis*. (**1**) 1,3,7-hydroxy xanthone, F137, and two geranyloxy xanthones, including (**2**) 7-geranyloxy-1,3-dihydroxy xanthone, F6, and (**3**) 3-geranyloxy-1, 7-dihydroxy xanthone, F8.

In this study, three new hydroxy xanthones, i.e., two geranyloxy xanthones (F6, F8) and a 1,3,7-hydroxy xanthone (F137) (Figure 1), isolated from the medicinal plant *C. cochinchinensis* were investigated. The antioxidant activity of the aqueous extract was determined using a colorimetric assay. The cytotoxicity and antioxidant effect of each hydroxy xanthone on PDLSCs were evaluated. The effects of these compounds on the Nrf2/HO-1 pathway and the osteogenic differentiation of PDLSCs under ROS-induced conditions were also investigated.

## 2. Results

### 2.1. Antioxidant Activity of C. cochinchinensis Derivatives

**Figure 2:**
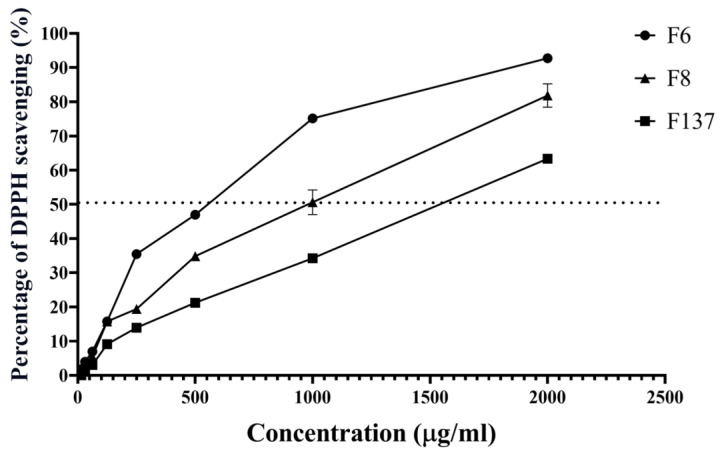
Radical scavenging effects of the *C. cochinchinensis* derivatives. F6 and F8 are geranyloxy xanthones, and F137 is a 1,3,7-hydroxy xanthone. Each experiment was performed three times.

The three pure compounds derived from *C. cochinchinensis* scavenged DPPH free radicals, and F6 demonstrated the lowest concentration (658 µg/mL) to decrease DPPH by 50%. In contrast, the concentrations of 1074 and 1543 µg/mL of F8 and F137, respectively, were required to scavenge the same amount of DPPH (Figure 2).

### 2.2. Cytotoxicity and ROS Production of C. cochinchinensis Derivatives in PDLSCs

**Figure 3:**
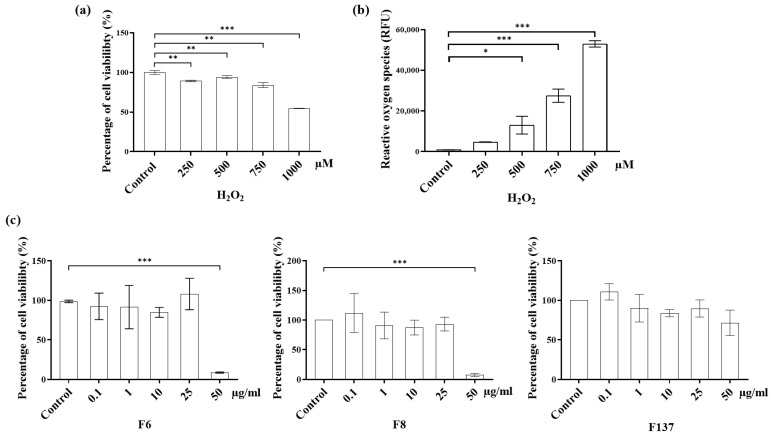
Cytotoxicity and reactive oxygen species (ROS) production. (**a**) Hydrogen peroxide (H_2_O_2_) demonstrated cytotoxicity and (**b**) stimulated ROS production in periodontal ligament stem cells. (**c**) The three *C. cochinchinensis* derivatives, F6, F8, and F137, did not demonstrate cytotoxicity at concentrations below 50 µg/mL. F6 and F8 are geranyloxy xanthones, and F137 is a 1,3,7-hydroxy xanthone. Each experiment was performed three times. *, **, and *** indicate significance at *p* < 0.05, 0.001, and 0.0001, respectively.

The results of cytotoxicity and intrinsic ROS stimulated by H_2_O_2_ demonstrate that higher H_2_O_2_ concentrations led to greater toxicity and intracellular oxidative stress (Figure 3). Less than 50% PDLSCs viability was observed when the H_2_O_2_ concentration reached 1000 mM. Moreover, the amount of free radical oxygen molecules also increased in a H_2_O_2_ concentration-dependent manner. In contrast, the three *C. cochinchinensis* derivatives did not exhibit cytotoxicity at concentrations below 50 µg/mL. The F137 treatment was not cytotoxic at any concentration. In contrast, the F6 and F8 derivatives were toxic to PDLSCs at concentrations ≥ 50 µg/mL.

### 2.3. Antioxidant Effects of C. cochinchinensis Derivatives

**Figure 4:**
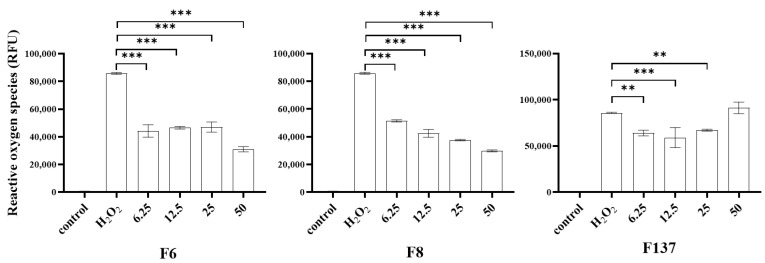
Antioxidant effects of the *C. cochinchinensis* derivatives on oxidative stress-induced periodontal ligament stem cells. H_2_O_2_-induced oxidative stress resulted in increased intrinsic reactive oxygen species, while combining the treatment with the *C. cochinchinensis* derivatives, F6, F8, and F137, demonstrated the opposite effect. F6 and F8 are geranyloxy xanthones, and F137 is a 1,3,7-hydroxy xanthone. Each experiment was performed three times. **, and *** indicate significant differences at *p* < 0.05, 0.001, and 0.0001, respectively.

The ROS levels were measured after treating PDLSCs with an oxidizer or antioxidant (Figure 4). The treatment with H_2_O_2_ alone activated the intrinsic ROS, and the treatment with H_2_O_2_ together with each *C. cochinchinensis* compound exhibited a reverse effect. At 6.25–50 ug/mL of each compound, PDLSCs demonstrated significantly reduced intrinsic ROS. Only the F137 derivative at 50 µg/mL did not have a significant antioxidant effect.

### 2.4. Effects of C. cochinchinensis Derivatives on HO-1 and Nrf2 Expression in PDLSCs

**Figure 5:**
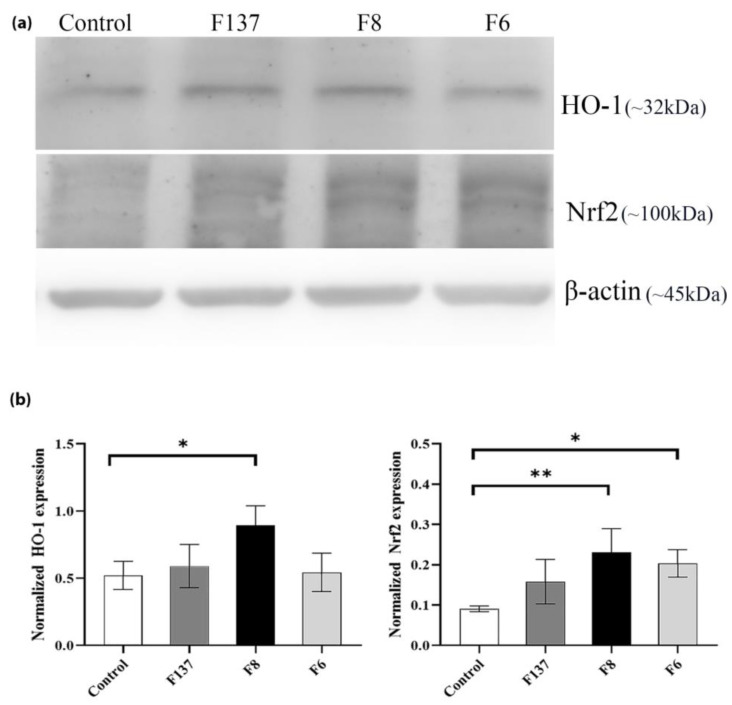
Effects of the *C. cochinchinensis* derivatives on HO-1 and Nrf2 expression in PDLSCs. (**a**) The three derivatives, F137, F8, and F6, at 25 µg/mL upregulated HO-1 and Nrf2 expression in PDLSCs. (**b**) HO-1 and Nrf2 were significantly increased in cells treated with F8. * and ** indicate significance at *p* < 0.05 and 0.001, respectively. F6 and F8 are geranyloxy xanthones, and F137 is a 1,3,7-hydroxy xanthone. Each experiment was performed three times.

The Western blot analysis demonstrated that the *C. cochinchinensis* derivatives upregulated HO-1 and Nrf2 expression in PDLSCs. However, significant increases in HO-1 and Nrf2 expression were only observed in PDLSCs treated with F8 compared with the control (Figure 5). Treatment with the F6 compound significantly increased Nrf2 expression compared with the control. In contrast, HO-1 expression slightly increased. Notably, F137 did not induce any significant protein upregulation.

### 2.5. Effects of C. cochinchinensis Derivatives on PDLSCs Osteogenic Differentiation

**Figure 6:**
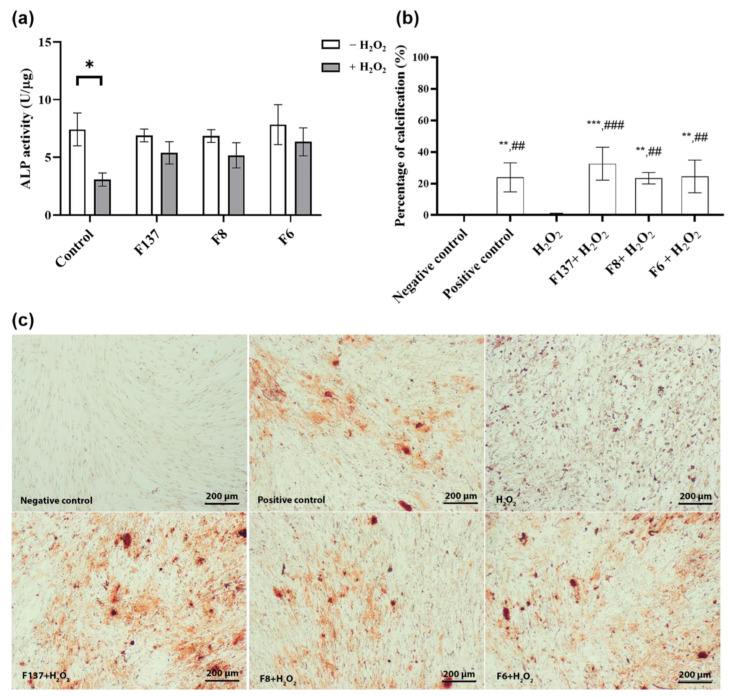
PDLSCs osteogenic differentiation after being treated with H_2_O_2_ and the three *C. cochinchinensis* derivatives for 7 and 21 days. (**a**) ALP activity evaluation after 7 days demonstrated that H_2_O_2_ treatment decreased ALP activity and that the treatment with each derivative and an oxidizing agent, H_2_O_2_, enhanced ALP activity. (**b**) The mineralization assay after 21 days demonstrated that each derivative and the positive control exhibited significantly more calcified nodules compared with H_2_O_2_ treatment. (**c**) During calcification, H_2_O_2_ impeded the formation of calcified nodules, while the xanthone derivative treatments improved the osteogenic differentiation and mineralization of PDLSCs under H_2_0_2_-induced oxidative stress. Each experiment was performed three times. ALP activity was analyzed using the multiple *t*-test, and a *p*-value ≤ 0.05 was considered significant. Percent mineralization was analyzed with one-way analysis of variance (ANOVA) followed by Turkey’s multiple comparisons. *,**^/##^, ***^/###^ indicate significance with H_2_O_2_ (*) or negative control (#) at *p* < 0.05, 0.001 and 0.0001, respectively.

The three *C. cochinchinensis* derivatives demonstrated antioxidant effects during PDLSCs osteogenic differentiation under stress induced by H_2_O_2_. Lower ALP activity was observed when PDLSCs were treated with H_2_O_2_ (Figure 6a), and each *C. cochinchinensis* extract improved the ALP activity of PDLSCs subject to H_2_O_2_-induced oxidative stress. In the late stage of mineralization (Figure 6b,c), H_2_O_2_ significantly reduced PDLSCs mineralization, and the percent calcification of the H_2_O_2_-treated group was similar to the negative control group (cells cultured in growth medium). In contrast, H_2_O_2_ together with each *C. cochinchinensis* derivative and the positive control revealed more calcified nodules compared with H_2_O_2_ alone.

## 3. Discussion

Periodontal disease is characterized by chronic bacterial accumulation and the subsequent expression of proinflammatory cytokines and the production of free radicals [5,17]. The ROS produced by polymorphonucleocytes as a part of the cellular defense mechanism should be eliminated quickly to reduce their harmful effects on the periodontal tissues. Various xanthone compounds isolated from *C. cochinchinensis* have demonstrated antioxidant effects and low cytotoxicity to human gingival fibroblasts [11]. In the present study, three xanthones isolated from *C. cochinchinensis* were investigated for their effects on PDLSCs, which are the cells principally responsible for regenerating the periodontium during periodontal disease. Our study revealed that the three hydroxy xanthones were effective ROS scavengers and that their effects on PDLSCs under redox conditions in terms of antioxidation promoted osteogenic differentiation. The three derivatives, i.e., F6, F8, and F137, demonstrated antioxidant effects at different concentrations. None of the derivatives were cytotoxic. Furthermore, the derivatives reduced intracellular ROS by upregulating HO-1 and Nrf-2 expression in PDLSCs. Although PDLSCs treated with F8 had the highest protein expression, the F137 treatment had the best antioxidant efficiency during osteogenic differentiation.

*C. cochinchinensis* species have been found in various areas across Southeast Asia, and their phytochemical components have shown antioxidant effects in numerous previous studies [11,18,19]. The three derivatives investigated in this study are stereoisomers of hydroxyxanthone with or without a geranyl group. We named the compounds F6, F8, and F137 and observed the percent inhibition of DPPH radicals. Although F137 has the most –OH groups in its chemical structure, its DPPH scavenging activity was lower compared with F6 and F8. This might have been due to the limitations of the DPPH assay, which cannot differentiate the radical quenching mechanism during the experiment, and in the DPPH assay, an electron transfer mechanism is more prone to occur compared with a hydrogen atom transfer one [20]. Furthermore, using methanol as the solvent in this assay might bind to hydrogen atoms and block radical neutralization [21]. These factors limit the chemical accuracy of this assay and its ability to predict which antioxidant is the most effective. Therefore, this study further investigated the antioxidant effects on the cellular behavior during PDLSCs’ exposure to free radicals and osteogenic differentiation that mimic the clinical condition.

Hydrogen peroxide is an oxidizing agent that is widely used as a stimulator in oxidative stress models. Intracellular damage occurs following the breakdown of hydrogen peroxide to ROS [22]. Although cells can neutralize the harmful effect from the free radicals, an excessive amount of the oxidizing agent can lead to cell death [22]. Our results demonstrate that hydrogen peroxide activated intracellular ROS production and increased cytotoxicity in a dose-dependent manner. In contrast, none of the *C. cochinchinensis* derivatives demonstrated cell toxicity below 50 µg/mL. Furthermore, the hydroxy xanthones acted as scavengers, and the pure compounds also inhibited ROS production in the H_2_O_2_-induced PDLSCs model. The three bioactive xanthones isolated from *C. cochinchinensis* have two benzene rings fused at different positions, and different numbers and positions of the hydroxyl group, which can affect the activity and potency of the compounds [16,23,24]. The xanthone derivatives have two mechanisms for scavenging free radicals, which are hydrogen atom transfer and single-electron transfer via proton transfer [25]. Furthermore, their antioxidant effects might be influenced by the number of phenol moieties in their chemical structure, the substitution position, H bonding, and whether the functional group is an electron-donating or electron-withdrawing group [24,26,27]. Based on its chemical structure, F137 was expected to show the greatest antioxidant effect. However, the intracellular ROS measurement results indicated that the three derivatives had a similar antioxidant effect, despite possessing different chemical structures. This finding might be due to other factors, such as bond-association enthalpy between hydrogen atoms and oxygen atoms or the ionization potential of the different atoms. These are the essential parameters for evaluating the role of an antioxidant and should be further investigated [23,26].

The antioxidant pathway of xanthone compounds involves their ability to neutralize free radicals and reduce oxidative stress. A general pathway includes scavenging free radicals, metal chelation, antioxidant-enzyme induction, reduced inflammation, and cellular signaling through the modulation of transcription factors like Nrf2. Nrf2/Keap1 signaling is a complex and multifaceted pathway that has wide-ranging effects on cellular and physiological processes. Its modulation has the potential to impact a variety of health conditions, such as cancer and inflammatory diseases [28,29,30,31]. The Western blot analysis of PDLSCs treated with the F8 xanthone derivative demonstrated significantly increased HO-1 and Nrf2 expression. HO-1 is one of the most critical cytoprotecting proteins [32], providing an adaptive cellular response against the toxicity of oxidative stress [33,34]. In an animal model, HO-1 upregulation attenuated complement-dependent inflammation, whereas its decrease led to the development of inflammation [35,36]. Nrf2 is a key transcription factor that regulates antioxidant protein expression to maintain cellular redox homeostasis [37,38]. This may explain why increased Nrf2 levels were found in our study. A previous study found that Nrf2 contributes to bone homeostasis and that decreased Nrf2 levels resulted in increased local oxidative damage and alveolar bone loss in chronic periodontitis [38]. HO-1 and Nrf2 upregulation maintain antioxidant/oxidant homeostasis [39], which might explain the reduction in ROS observed in our study. However, F6 alone upregulated Nrf2 expression without a substantial increase in HO-1, indicating potential antioxidant effects through alternative Nrf2-dependent protein pathways, e.g., NAD(P)H:quinone oxidoreductase 1 and glutamate–cysteine ligase. Conversely, F137 did not induce a significant increase in either Nrf2 or HO-1 expression, suggesting the involvement of an alternative cell signaling pathway that requires further investigation. Furthermore, although our study provides valuable insights into the antioxidant pathways of xanthone compounds and their impact on HO-1 and Nrf2 expression, we acknowledge that this research may leave questions about the specific mechanisms involved. Additional studies in this area could provide a more comprehensive understanding of the pathways, revealing additional redox-sensitive transcription factors and targets, such as nuclear factor-κB and mitogen-activated protein kinase, that may contribute to *C. cochinchinensis*-mediated antioxidant effects.

Bone regeneration is the primary objective in treating periodontal disease, with ROS generated by inflammatory cells impeding PDLSCs efficiency [4,5]. Our overall results suggest that *C. cochinchinensis* derivatives are promising agents to slow periodontal tissue destruction in the context of excessive redox conditions during inflammation. Thus, the present study demonstrates the potential of these pure compounds as antioxidants during bone regeneration. In our experimental model, PDLSCs were stressed using a simulated H_2_O_2_ redox condition, and the cells demonstrated decreased ALP activity and calcification. In addition, hydroxy xanthone treatment reduced the effect of the oxidative stress induced by H_2_O_2_. Among the three derivatives, F137 generated the highest increase in percent calcification. This finding suggests that the osteogenic environment might be appropriate for the activity of F137, which has many –OH groups and does not have a geranyl group. Accordingly, *C. cochinchinensis* derivatives, especially F137, could be used as effective antioxidants during bone regeneration.

## 4. Materials and Methods

### 4.1. Extraction and Isolation of C. cochinchinensis Derivatives

The plant material was cultivated and collected in Kaun Kha Long district, Satun Province, Southern part of Thailand, in October 2007 and was identified as the young fruit of *Cratoxylum cochinchinens* by Prof. Puangpen Sirirugsa from Prince of Songkla University, complying with the IUCN Policy Statement on Research Involving Species at Risk of Extinction and the Convention on the Trade in Endangered Species of Wild Fauna and Flora. A voucher specimen (No. SL-1) was deposited in the herbarium of the Department of Biology, Prince of Songkla University, Songkhla, Thailand, and it was allowed to be used in this study. The air-dried green fruit of *C. cochinchinense* was extracted with Dichloromethane (CH_2_Cl_2_) through maceration at room temperature and was further evaporated under reduced pressure to obtain a deep-green, crude CH_2_Cl_2_ extract. The crude CH_2_Cl_2_ extract was fractionated on a silica gel QCC using hexane as the first eluent and increasing polarity with ethyl acetate to generate nine fractions. Fraction six was further separated using column chromatography (CC) and eluted with pure CHCl_3_ to give compounds F8 and F6, respectively. Fraction nine was further purified using CC and eluted with an acetone–hexane gradient to give compound F137. Each of the isolated compounds was confirmed using Nuclear Magnetic Resonance spectroscopy (Supplementary Data S1) and lyophilized to produce powder that was stored in a closed tube in a desiccator at room temperature.

### 4.2. Antioxidant Activity of C. cochinchinensis Derivatives

The antioxidant activity the *C. cochinchinensis* derivatives was determined using a colorimetric assay. A volume of 100 μl of 60 µM 1-diphenyl-2-picrylhydrazyl (α,α-diphenyl-β-picrylhydrazyl; DPPH) (Sigma Aldrich, St. Louis, MO, USA) in absolute ethanol was added to the extracts, which were serially diluted in methanol from 3 mg/mL to 30 µg/mL. An equal amount of each sample was loaded into the wells of a 96-well plate, followed by mixing with 100 µL of DPPH solution before incubation in the dark for 30 min. The colorimetric change was measured before and after incubation using a microplate reader (BioTek Model series UV 900Hdi; Winooski, VT, USA) at 515 nm. Percent radical scavenging was determined using the following equation:

$$\%\;\mathrm{radical}\;\mathrm{scavenging}=\left(\left(\mathrm{Ac}-\left(\Delta \mathrm{As}\right)\right)/\mathrm{Ac}\right)\times 100$$

where Ac is the absorbance of the control loaded only with DPPH solution and ΔAs is the absorbance difference of the solution containing the plant extract and DPPH between before and 30 min after the test.

### 4.3. PDLSCs Isolation and Characterization

PDLSCs were isolated from sound premolars or molars of healthy 15–20-year-old patients. The teeth were extracted due to reasons unrelated to this study at the Oral and Maxillofacial surgery clinic. All procedures were in accordance with the ethical standards of the Institutional Review Board of the Faculty of Dentistry/Pharmacy, Mahidol University (COA No. MU-DT/PY-IRB 2017/048.0611) and with the 1964 Helsinki Declaration and its later amendments or comparable ethical standards. Mesenchymal stem cell characterization was performed in a previous study [40].

### 4.4. Cytotoxicity and ROS Production of C. cochinchinensis Derivatives in PDLSCs

The *C. cochinchinensis* derivatives were tested for their toxicity against PDLSCs in parallel with a H_2_O_2_ solution (control) using a colorimetric assay. A total of 10^5^ cells per well were cultured in a 24-well plate for 24 h in α-MEM supplemented with 10% FBS and 1% penicillin/streptomycin (growth medium) prior to the test. Each *C. cochinchinensis* derivative was used at 50, 25, 10, 1, and 0.1 µg/mL, and the H_2_O_2_ solution was tested at 200, 500, 750, and 1000 µM. Once the cells were confluent, the test solution was loaded into each well, and cells were cultured at 37 °C in a 5% CO_2_ chamber. Percentage cell viability was analyzed using methylthiazolyldiphenyl-tetrazolium bromide (MTT; Sigma-Aldrich, St. Louis, MO, USA) according to the manufacturer’s instructions. The test solutions were evaluated in triplicate, and the percentage cell viability was calculated from the absorbance measured at 570 nm and 690 nm compared with the control group. The intracellular ROS following treatment with H_2_O_2_ solution was determined in parallel using 5-(and-6)-chloromethyl-2′,7′-dichlorodihydro-fluorescein diacetate, ROS dye (Invitrogen, Waltham, MA, USA), according to the manufacturer’s instructions. Fluorescence was measured with a microplate reader at excitation/emission spectra of 530/488 nm.

### 4.5. Effects of Cratoxylum cochinchinensis Derivatives on Oxidative Stress

The effects of the *C. cochinchinensis* derivatives on oxidative stress were determined in PDLSCs based on the reduction of intracellular ROS after H_2_O_2_ stimulation. PDLSCs (10^4^ cells/well) were cultured in a 96-well plate with growth medium containing 750 µM of H_2_O_2_ and *C. cochinchinensis* derivatives at 50, 25, 12.5, and 6.25 µg/mL for 4 h at 37 °C in a 5% CO_2_ chamber. ROS dye was added to each well, followed by fluorescence measurement in a microplate reader at excitation/emission spectra of 530/488 nm. Wells without cells were used as blanks, and the test solutions were evaluated in triplicate.

### 4.6. Western Blot Analysis of HO-1 and Nrf2 Expression in PDLSCs

The protein expression of HO-1 and Nrf2 was investigated using the Western blot technique. PDLSCs (10^6^ cells in T25 flasks) were treated with 25 µg/mL of *C. cochinchinensis* derivatives or growth medium (control) for 1 h, and the treated cells were washed with PBS twice before we extracted cellular proteins with protease inhibitor cocktail (Thermo^®^ Fischer Scientific, Waltham, MA, USA); then, the total protein concentrations were determined. A total of 20 μg of protein extracts was separated using 10% polyacrylamide gel, and Western blot was performed. The separated proteins were transferred to a 0.2 µm polyvinylidene difluoride membrane using transfer buffer containing 25 mM Tris, 192 mM glycine, and 20% methanol at pH 8.3 and ambient temperature using 220 mA for 2 h. The membranes were washed with Tris-buffered saline (TBS) before being blocked with 5% non-fat milk in TBS combined with 0.1% Tween20. The membranes were subsequently incubated with primary antibodies, anti-HO-1 (1:250; ab13248; Abcam, Cambridge, UK), anti-β-actin (1:1,000; #4970; Cell Signaling, Danvers, MA, USA), or anti-Nrf2 (1:200; ab62352; Abcam) antibody, and detected with a secondary antibody using goat anti-rabbit IgG (ab6721; Abcam) or rabbit anti-mouse IgG conjugated to horseradish peroxidase (ab6728; Abcam). The protein bands were analyzed using enhanced chemiluminescence (Bio-Rad, Hercules, CA, USA), and images were obtained using a biomolecular imager (Cytiva; Marlborough, MA, USA).

### 4.7. Effects of C. cochinchinensis Derivatives on PDLSCs Osteogenic Differentiation

PDLSCs (10^5^ cells/well) were seeded in a 24-well plate and incubated with osteogenic medium consisting of 0.1 μM dexamethasone (Sigma-Aldrich), 50 μg/mL ascorbate-2-phosphate (Sigma-Aldrich), 10 mM β-glycerophosphate (Sigma-Aldrich) in growth medium with or without 1000 µM H_2_O_2_. *C. cochinchinensis* derivatives (6.25 µg/mL) were added to the osteogenic medium two days prior to loading H_2_O_2_. The control was osteogenic medium. Alkaline phosphatase (ALP) activity was detected on day 7 after osteogenic induction using a colorimetric assay kit (Abcam). Briefly, the cells were washed twice with PBS and loaded with lytic buffer for 15 min before being removed using a cell scraper according to the manufacturer’s recommendations. The cell lysate was centrifuged at 10,000× *g* for 10 min, and 80 µL of supernatant was mixed with 50 µL of 5 mM *p*-nitrophenylphosphate (pNPP) as the substrate for 1 h at 37 °C. The reaction was stopped, and the absorbance was measured at 405 nm. Total proteins were quantified using a BCA kit (Sigma). Enzyme activity was expressed as µmol/min/µg or U/µg. The PDLSCs were treated with each *C. cochinchinensis* derivative together with H_2_O_2_ in parallel with the group treated with only H_2_O_2_ for 21 days. Alizarin Red staining was used to stain the calcified nodules, and the stained calcified nodules and percent calcification were quantified using ImageJ as previously described [36]. The positive control consisted of PDLSCs cultured in osteogenic medium, and the negative control consisted of PDLSCs cultured in growth medium.

### 4.8. Statistical Analysis

The normality of the data was determined before further statistical analyses. Cytotoxicity, ROS production, protein expression, and percent mineralization were analyzed with one-way analysis of variance (ANOVA), followed by Turkey’s multiple comparisons. ALP activity was analyzed using the multiple *t*-test, and a *p*-value ≤ 0.05 was considered statistically significant difference.

## 5. Conclusions

We have presented extensive data on the effects of hydroxy xanthones from *C. cochinchinensis* on osteogenic differentiation, antioxidation, and the host inflammatory response that involves the activation of the stress response Nrf2/HO-1 pathway. Using these compounds may activate the intrinsic cellular mechanism of PDLSCs to survive to the effects of the inflammatory cells while stimulating periodontium regeneration. These compounds could become an alternative for periodontal treatment that is effective, safe, and sustainable.

## Data Availability

Data are contained within the article or Supplementary Materials.

## Supplementary Materials

The following supporting information can be downloaded at: https://www.mdpi.com/article/10.3390/ijms241914675/s1.
