# Supplementary material for: Xanthones Isolated from Cratoxylum cochinchinensis Reduced Oxidative Stress in Periodontal Ligament Stem Cells
Source: Int J Mol Sci. 2023 Sep 28;24(19):14675. doi: 10.3390/ijms241914675 (PMC10573000; doi:10.3390/ijms241914675)
Supplement: Supplementary file 1 [file ijms-24-14675-s001.zip › ijms-2623913-supplementary.pdf]

1,3,7-trihydroxyxanthone (**1**). Yellow powder, mp = 318-319 °C. For ^{1}H NMR (300 MHz, CD_3OD + $CDCl_3$) spectroscopic data: δ 12.92 (s), 7.51 (d, J = 2.7 Hz, H-8), 7.33 (d, J = 9.0 Hz, H-5), 7.25 (dd, J = 9.0, 2.7 Hz, H-6), 6.37 (d, J = 2.1 Hz, H-4) and 6.25 (d, J = 2.1 Hz, H-2).

7-geranyloxy-1,3-dihydroxyxanthone (**2**). Yellow powder, m.p. 138- 140 °C; UV-Vis λ_{max} (log ε) 206 (4.25), 236 (4.48), 260 (4.39), 316 (3.89), 364 (3.94) nm; FT-IR (KBr) ν_{max} 3162, 1652 cm^{-1}. For ^{1}H NMR (300 MHz, $CDCl_3$) spectroscopic data: δ 12.85 (s), 7.86 (br *s*, 3-OH), 7.40 (br d, J = 1.5 Hz, H-8), 7.18 (br d, J = 9.6 Hz, H-6), 7.14 (br d, J = 9.0 Hz, H-5), 6.27 (d, J = 1.8 Hz, H-4), 6.22 (d, J = 1.8 Hz, H-2), 5.39 (br t, J = 6.3 Hz, H-2′), 4.99 (br t, J = 5.7 Hz, H_2-6′), 4.45 (d, J = 6.3 Hz, H_2-1′), 2.01 (*m*, H_2-4′), 1.95 (*m*, H_2-5′), 1.64 (*s*, H_3-9′), 1.57 (*s*, H_3-8′) and 1.50 (*s*, H_3-10′).

3-oxygeranyl-1,7-dihydroxyxanthone (**3**). Yellow powder, m.p. 147-148 °C; UV-Vis ($CHCl_3$) λ_{max} (log ε) 203 (4.49), 229 (4.30), 259 (4.31), 307 (3.99), 374 (3.63) nm; FT-IR (KBr) ν_{max} 3288, 1647 cm^{-1}. For ^{1}H (300 MHz, $CDCl_3$) spectroscopic data: δ 12.73 (s), 7.40 (br s, H-8), 7.30 (br d, J = 9.3 Hz, H-5), 7.26 (br d, J = 9.3 Hz, H-6), 7.03 (br *s*, 7-OH), 6.40 (d, J = 2.4 Hz, H-4), 6.34 (d, J = 2.4 Hz, H-2), 5.50 (br t, J = 6.6 Hz, H-2′), 5.11 (br t, J = 5.7 Hz, H_2-6′), 4.63 (d, J = 6.6 Hz, H_2-1′), 2.14 (*m*, H_2-4′), 2.10 (*m*, H_2-5′), 1.78 (*s*, H_3-9′), 1.69 (*s*, H_3-8′) and 1.63 (*s*, H_3-10′).
